# Respiratory Mitochondrial Efficiency and DNA Oxidation in Human Sperm after In Vitro Myo-Inositol Treatment

**DOI:** 10.3390/jcm9061638

**Published:** 2020-05-28

**Authors:** Laura Governini, Rosetta Ponchia, Paolo Giovanni Artini, Elena Casarosa, Ilaria Marzi, Angela Capaldo, Alice Luddi, Paola Piomboni

**Affiliations:** 1 Department of Molecular and Developmental Medicine, Siena University, 53100 Siena, Italy; laura.governini@unisi.it (L.G.); ponchia2@student.unisi.it (R.P.); piomboni@unisi.it (P.P.); 2 Assisted Reproduction Unit, Siena University Hospital, 53100 Siena, Italy; angela.ca@live.it; 3 Department of Experimental and Clinical Medicine, Division of Obstetrics and Gynecology, Pisa University, 56100 Pisa, Italy; pgartini@gmail.com (P.G.A.); elena.casarosa@dmdp.unipi.it (E.C.); ilaria.marzi1@gmail.com (I.M.)

**Keywords:** human sperm, myo-inositol, sperm motility, DNA oxidation, mitochondrial respiration

## Abstract

Semen samples are known to contain abnormal amounts of reactive oxygen species (ROS) and oxygen free radicals; therefore, the identification of antioxidant molecules able to counteract the oxidative damage caused by ROS is foresight. Indeed, improving semen quality in terms of motility and reduction in DNA damage, can significantly improve the fertilization potential of sperm in vitro. To this regard, myo-inositol, based on its antioxidant properties, has been reported to be effective in improving sperm quality and motility in oligoasthenozoospermic patients undergoing assisted reproduction techniques when used as a dietary supplementation. Moreover, in vitro treatment demonstrated a direct relationship between myo-inositol, mitochondrial membrane potential and sperm motility. This experimental study aimed to evaluate the effects of myo-inositol (Andrositol-lab) in vitro treatment on sperm motility, capacitation, mitochondrial oxidative phosphorylation and DNA damage. Our results demonstrate that myo-inositol induces a significant increase in sperm motility and in oxygen consumption, the main index of oxidative phosphorylation efficiency and ATP production, both in basal and in in vitro capacitated samples. Moreover, we provide evidence for a significant protective role of myo-inositol against oxidative damage to DNA, thus supporting the in vitro use of myo-inositol in assisted reproductive techniques. Even if further studies are needed to clarify the mechanisms underlying the antioxidant properties of myo-inositol, the present findings significantly extend our knowledge on human male fertility and pave the way to the definition of evidence-based guidelines, aiming to improve the in vitro procedure currently used in ART laboratory for sperm selection.

## 1. Introduction

Male infertility represents an urgent social burden, considering that 15% of all couples around the world are infertile and that male factor can be diagnosed in about 50% of these cases [1,2,3]. The most frequent and typical causes for male infertility are represented by varicocele, cryptorchidism, infections, obstructive lesions, trauma, and cancer [1]; noteworthily, the exact etiology of male factor infertility still remains undiagnosed in about 30% to 50% of patients, who therefore are classified as idiopathic cases [4]. There is increasing evidence that oxidative stress (OS) represents a condition having a key role in the etiology of male infertility and thus it has been studied extensively [5,6].

OS is defined as a result of the imbalance between reactive oxygen species (ROS) and antioxidants; the presence of anomalous concentrations of ROS has been detected in 30% to 80% of infertile men and this condition seems to lead to sperm damage, and eventually male infertility [7,8]. ROS are the most widespread group of free radicals that, due to an unpaired electron in the outer orbital, may trigger an extremely detrimental chain mechanism mainly affecting both the plasma membrane and DNA [9]. Based on this mechanism, ROS are capable of compromising sperm quality and functions.

The main endogenous sources of ROS in seminal fluid are represented by leukocytes, especially neutrophils, as well as mitochondria and plasma membranes of morphologically abnormal sperm [10,11,12]. ROS production is normally present in physiologic conditions; indeed, neutrophils are always released into secretions from the prostatic and seminal vesicle. This physiologic production is crucial for spermatogenesis and fertility, since ROS has been reported to be fundamental for sperm motility and viability as well as in sperm maturation, hyperactivation, capacitation and acrosome reaction [13,14,15]. On the other end, pathologic conditions such as inflammation or infections may induce an overproduction of proinflammatory molecules (cytokines and interleukins) that, in turn, may increase ROS production [10,16]. This increase gives rise to an imbalance between ROS generation and antioxidant capacity in favor of the oxidants, thus establishing an oxidative stress condition that negatively affects sperm morphology and functions through lipid peroxidation, DNA fragmentation, and apoptosis [17,18,19]. Noteworthily, the high polyunsaturated fatty acid content of sperm plasma membranes, that easily may undergo lipid peroxidation, makes sperm very susceptible to OS damage. Indeed, lipid oxidation causes an increase in the permeability of both the plasma membrane and mitochondrial membrane with the resulting decrease in motility and increased cell apoptosis [17,20]. On the other end, mitochondrial DNA (mtDNA) is extremely sensitive to damage from radicals due to the lack of conventional histone proteins and limited mitochondrial damage repair capability; therefore, the resulting accumulation of mtDNA mutations can affect spermatogenetic activity, also causing oligoasthenozoospermia, one of the main causes of male infertility characterized by a reduced number of spermatozoa in the ejaculate and low cell motility, reduced fertilizing capacity and spontaneous abortions in the first months of gestation [21,22]. The 8-Hydroxy-2′-deoxyguanosine (8-OHdG) is known as the early products of oxidative DNA damage [23] and, for this reason, it has been used as a specific and quantitative biomarker for oxidative stress [24,25]. Indeed, many studies demonstrate the existence of a close correlations between 8-OHdG level and sperm morphology and functionality, thus supporting the potential diagnostic value of this assay in clinical practice [26,27].

In light of the here described detrimental effects of oxidative stress on sperm, an antioxidant able to prevent oxidative damage and improve sperm motility could be beneficial in the management of male infertility. In this regard, non-enzymatic antioxidants, both synthetic and dietary supplements, are considered a significant therapeutic option. Between others, inositols are cyclic carbohydrates existing in nine stereoisomeric forms: myo-inositol is the most abundant form in nature. Several studies have shown that myo-inositol has powerful antioxidant properties that improve sperm quality, particularly in patients with oligoasthenozoospermia [28,29,30,31].

In male reproductive organs, myo-inositol has a central physiologic role; indeed, it is mainly produced by Sertoli cells in response to follicle-stimulating hormone (FSH) and is involved in processes including the regulation of motility, capacitation and acrosome reaction of sperm cell [28,29,32]. Moreover, its concentrations increase from the epididymis to the deferent duct; in fact, myo-inositol would seem to play an important role in the success of spermatogenesis and spermio-histogenesis as well as in osmoregulation of seminal vesicular fluid, contributing to reduce the viscosity [33] and the presence of amorphous material [34] in seminal fluid. Moreover, myo-inositol is involved in the control of intracellular Ca^2+^ concentration, gene expression, cytoskeleton assembly, inhibition of mechanisms that mediate cell death by apoptosis and in maintaining the mitochondrial membrane potential (MMP) [30]. Through the sophisticated mechanism of the oxidative phosphorylation system (OXPHOS), mitochondria play a fundamental role in the production of ATP necessary for sperm motility, capacitation, hyperactivation and acrosome reaction [35]; anyway, mitochondria are also actively implicated in other important processes, including ROS generation [36].

In light of these observations, the purpose of this study was to test the efficacy of a myo-inositol-based product in in vitro ameliorating different sperm parameters, such as basal motility, vitality and sperm recovery after swim-up selection. Moreover, the effectiveness of myo-inositol in vitro treatment in counteracting oxidative stress damage to DNA, by means of 8-OHdG assay, has been undertaken. Finally, mitochondrial respiratory capacity of sperm has been analyzed by oxygraphic approach, in order to definitively provide evidence for a specific activity of myo-inositol in ameliorating the OXPHOS in human sperm.

## 2. Materials and Methods

### 2.1. Patients

This study was conducted on a total of 56 Caucasian males undergoing semen evaluation at the Unit of Medically Assisted Reproduction, Siena University Hospital and at Centre of Infertility and Assisted Reproduction of Pisa University Hospital. A comprehensive clinical history of patients was obtained; we excluded patients with possible causes of male infertility such as varicocele, cryptorchidism, endocrine disorders or systemic diseases and patients with intake of spermiotoxic drugs, smoking, alcohol or drugs abuse. The median age of the patients was 33 years (range: 25–45 years); the BMI ranged between 18 and 25. All participants signed a written informed consent, and the study protocol was approved by the Ethic Committeeof the Siena University Hospital (approval ID: CEASVE 191113).

### 2.2. Sample Treatment

The evaluation of the sperm parameters was carried out in accordance with the WHO (2010) criteria [37]. The ejaculate samples examined were collected after a period of abstinence variable from 2 to 5 days, by masturbation, in sterile containers. The evaluation of the seminal parameters was carried out, within 30 min after fluidification, by a blinded observer and repeated for quality control by another blinded observer (obtained data are the mean value of two observations). After an accurate resuspension of the sample, sperm concentration, progressive and non-progressive motility, and morphology were evaluated.

In order to measure the effect of myo-inositol in vitro treatment on sperm in basal condition, semen samples from 20 normozoospermic patients were divided into three aliquots, each containing about 20 million sperm. These aliquots were incubated at 37 °C for 30 min with: (i) standard medium (untreated sample), or medium supplemented with myo-inositol (Andrositol Lab, Lo.Li. Pharma, Rome, Italy) at (ii) 2 mg/mL and (iii) 20 mg/mL (treated sample). After in vitro treatment, sperm concentration and progressive motility were assessed before to proceed to subsequent analyses.

In order to measure the effect of myo-inositol in vitro treatment on sperm capacitation, semen samples from 16 normozoospermic patients were divided into two aliquots, each containing about 20 million sperm. These aliquots were incubated with standard medium (untreated sample) or medium supplemented with myo-inositol at 20 mg/mL (treated sample) for 20 min. After that, each aliquot was subjected to the in vitro capacitation by swim-up procedure. Briefly, the semen samples were mixed gently with the Quinn’s Advantage Medium and tubes were centrifuged at 260 g for 10 min. The supernatant was removed and the pellet was carefully overlaid with 0.8 mL of fresh Quinn’s Sperm Washing Medium and incubated 30 min at 37 °C; at the end, an upper layer (0.2 mL) was collected and, by using a Makler counting chamber (Irvine Scientific, Santa Ana, CA, USA), we assessed progressive motility and sperm.

### 2.3. Hypotonic Swelling and Oxygraphic Assay

After the in vitro treatment described above, sperm samples were centrifuged at 800 g for 10 min and then washed by resuspension in isotonic salt medium (113 mmol/L KCl, 12.5 mmol/L of KH_2_PO_4_, 2.5 mmol/L of K_2_HPO_4_, 3 mmol/L of MgCl_2_, 0.4 mmol/L of ethylenediaminetetraacetic acid or EDTA and 20 mmol/L of Tris adjusted to pH 7.4 with HCl). Sperm cells were then subjected to hypotonic treatment as described by Stendardi et al. [38]. Briefly, sperm cells were kept in ice-chilled hypotonic medium (potassium phosphate 10 mmol/L, pH 7.4, with 2 g/L of bovine serum albumin or BSA) for 1.5 h. Sperm were then washed three times using isotonic salt medium, pH 7.4 and, sperm concentration was adjusted to about 15 million for basal and 3 million for capacitated sperm samples to be used in each oxygraphic experiment.

Oxygen uptake by spermatozoa was measured at 36 °C using a Clark-type oxygen probe (HansatechOxygraph; Pentney, King’s Lynn, UK). Demembranated sperm cells were stirred vigorously in the reaction chamber (1 mL) in isotonic salt medium without EDTA and temperature equilibrated. The rate of oxygen uptake by spermatozoa (V) was expressed as nmol O_2_·mL^−1^·min^−1^. The oxygen consumed by the sperm cells was evaluated by calculating the difference between the oxygen present at the time of the addition of the spermatozoa and that present after 10 min.

### 2.4. Extraction of Genomic DNA and Measurement of DNA Oxidative Damages

Genomic DNA was extracted from 20 treated and 20 untreated samples. Briefly, sperm were centrifuged at 400 × g for 10 min, then the supernatant was removed. Cells were resuspended in 500 µL of lysis buffer (Tris-HCL 25 mM, pH 8.1, EDTA 5 mM, SDS 1%, proteinase K 0.4 mg/mL) and incubated at 55 °C for 6 h with gentle agitation. Sperm lysates were phenol/chloroform/isoamyl (25:24:1, pH8) extracted and the DNA recovered by ethanol precipitation in the presence of ammonium acetate and Glycogen. DNA precipitates were resuspended in TE buffer (Tris-HCl 10 mM, pH 8.1, EDTA 1 mM). Processed DNA samples were quantified by Nanodrop apparatus (Thermo Fisher Scientific) and stored in TE buffer at −80 °C.

The evaluation of oxidative stress in samples treated with myo-inositol versus not treated was performed by -8-hydroxy 2 deoxyguanosine Elisa kit (ab201734, Abcam, Cambridge, UK), according to the instruction for use included in the kit. All samples were tested in duplicate. Absorbance values were measured on a microplate reader.

### 2.5. Transmission Electron Microscopy

For Transmission Electron Microscopy (TEM), sperm samples in basal condition, in untreated or treated with myo-inositol were fixed in cold Karnovsky fixative and maintained at 4 °C for 2 h. Fixed semen was washed in 0.1 mol L^−1^ cacodylate buffer (pH 7.2) for 12 h, postfixed in 1% buffered osmium tetroxide for 1 h at 4 °C, dehydrated and embedded in Epon Araldite resin. Ultra-thin sections were cut with a Supernova ultramicrotome (Reickert Jung, Vienna, Austria), mounted on copper grids, stained with uranyl acetate and lead citrate and observed and photographed with transmission electron microscope (Tecnai G2 Spirit, FEI, Hillsboro, OR, USA) operating at an electron accelerating voltage of 120 kV equipped with a Morada (EMSIS) CCD camera.

### 2.6. Statistical Analysis

A statistical analysis was performed using GraphPad Prism 5.0 (GraphPad Software, La Jolla, CA, USA). The data are reported as mean ± SD. To detect differences between the control samples and those treated with myo-inositol, a student’s t-test was performed. Differences were considered statistically significant at *p* < 0.05. The results were reported as mean ± SD.

## 3. Results

### 3.1. Myo-inositol In Vitro Treatment Increases the Progressive Motility and the Oxygen Consumption Rate of Uncapacitated Sperm

**Figure 1:**
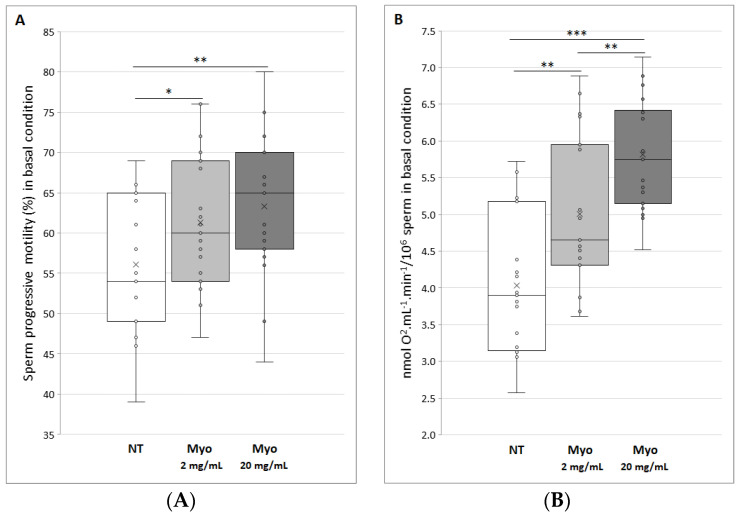
(**A**) Progressive motility of control spermatozoa (NT, not treated) and treated with myo-inositol (Myo) at a dose of 2 mg/mL (light gray) (*p < 0.05) and 20 mg/mL (dark gray) (**p < 0.01). (**B**) Oxygen consumption, express as nmol O_2_·mL^−1^·min^−1^ on 10 million sperm cells, of control spermatozoa (NT, not treated) and treated with myo-inositol (Myo) at a dose of 2 mg/mL (light gray) (**p < 0.01) and 20 mg/mL (dark gray) (***p < 0.001). Graphical diagrams are plotted as box–whisker plots, where boxes show the interquartile range with median and mean values, and whiskers represent min and max confidence intervals.

The first evidence we obtained when semen samples were in vitro treated with myo-inositol, was the complete absence of cell toxicity. Moreover, this compound was demonstrated to significantly affect the average progressive motility of sperm. In fact, as shown in Figure 1A, treatment with myo-inositol at a dose of 2 and 20 mg/mL determines a significant increase in progressive motility of 9% (*p* < 0.05) and 13% (*p* < 0.01), respectively.

After evaluating the effect of myo-inositol on sperm motility, we assessed oxygen consumption by oxygraphic analysis, as index of oxidative phosphorylation and, as a consequence, of ATP production. According to the increase in progressive motility measured following treatment with myo-inositol, the consumption of O_2_ by sperm reveals the same trend. Indeed, as shown in Figure 1B, treatment with myo-inositol at a dose of 2 and 20 mg/mL determines a significant dose-dependent increase in oxygen consumption of 24% (*p* < 0.01) and 44% (*p* < 0.001), respectively. Interestingly, the highest dose of myo-inositol increases about 1.3 times the oxygen consumption if compared to the lowest dose used (*p* < 0.01), that is, the dose currently suggested in the literature.

### 3.2. Myo-inositol Treatment Before In vitro Sperm Capacitation Increases Swim Up Sperm Recovery, Progressive Motility and the Oxygen Consumption Rate

**Figure 2:**
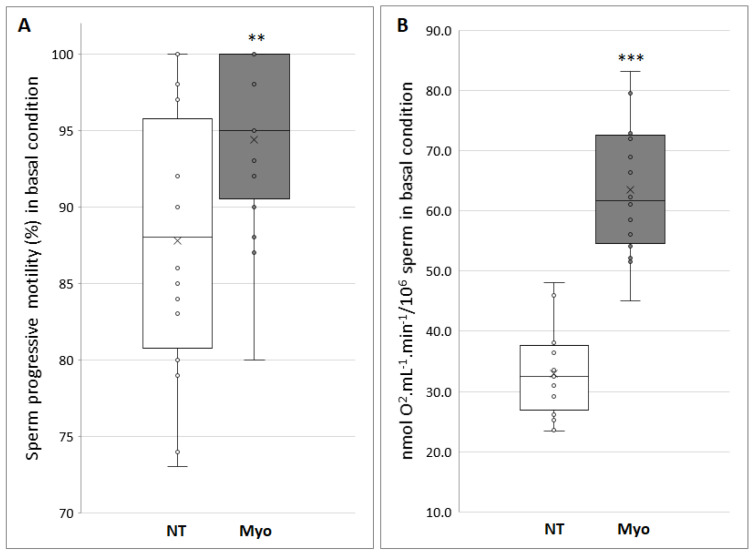
(**A**) Progressive motility of capacitated spermatozoa (NT, not treated) and treated with 20 mg/mL of myo-inositol (Myo) (**p < 0.01). (**B**) Oxygen consumption, expressed as nmol O_2_·mL^−1^·min^−1^ on 10 million sperm cells, of control spermatozoa (NT, not treated) respect to treatment with myo-inositol (Myo) at a dose of 20 mg/mL (***p < 0.001). Graphical diagrams are plotted as box–whisker plots, where boxes show the interquartile range with median and mean values, and whiskers represent min and max confidence intervals.

In order to assess whether myo-inositol treatment may be effective in ameliorating in vitro sperm capacitation procedures, we treated in vitro semen samples for 20 min with the most effective dose of myo-inositol (20 mg/mL) or of standard sperm medium, as a control. After that, semen underwent in vitro capacitation by means of a swim-up procedure. This approach enabled us to demonstrate that in vitro myo-inositol supplementation before swim-up guarantees a sperm recovery increase of about 35% (*p* < 0.001). Moreover, as shown in Figure 2A, a significant increase in progressive motility of about 10% may be observed (87.3% in untreated versus 94.8% treated samples; *p* < 0.01).

When oxygen consumption was assessed in swim-up selected sperm, we provide evidence for a two-fold increase (*p* < 0.001) in oxygen consumption in sperm selected after myo-inositol in vitro treatment (Figure 2B); this datum is in agreement with both the significant increase in recovered sperm and in progressive motility percentage, guaranteed by myo-inositol supplementation.

### 3.3. TEM Evaluation of Untreated and Treated Sample with Myo-inositol

**Figure 3:**
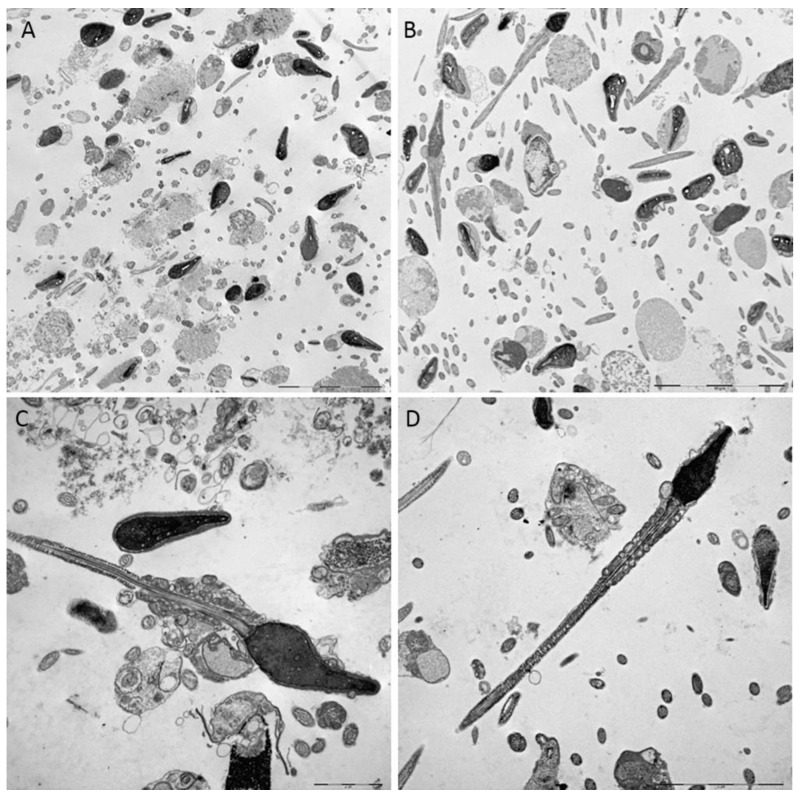
Transmission electron microscopy representative micrographs of sperm untreated (**A**) and (**C**); treated with myo-inositol (**B**) and (**D**). In the untreated samples, disperse flocculent material is evident around the spermatozoa; the treated samples do not show appreciable ultrastructural changes, but the surrounding environment appears extremely clean and many sperm with straightened tails are found in the same field of observation. (**A**–**B**) Bar = 10μm; (**C**–**D**): Bar = 2μm.

To characterize the sperm at ultrastructural level, semen samples, untreated or treated with myo-inositol at the dose of 20 mg/mL, were analyzed by TEM. The treated sperm did not show appreciable ultrastructural changes; however, it may be highlighted that treated sperm appear extremely “clean” from any amorphous fibrous material (Figure 3C–D) which instead was clearly evident on the surface and in the surrounding environment of the untreated sperm (Figure 3A–B). This could depend on the ability of myo-inositol to disrupt the mucoid masses of the seminal fluid, in agreement with what was claimed by Scarselli et al. [39]. Moreover, in treated samples, several sperm with uncurled tails with well-organized mitochondrial sheath can be frequently found in the same field of observation, that is an unusual finding at the electron microscopy level; it was evident that in the straightened tails, mitochondrial cristae appeared expanse and more electrondense.

### 3.4. The Use of 8-hydroxy-2′-deoxyguanosine as a Marker of Oxidative Damage to DNA

**Figure 4:**
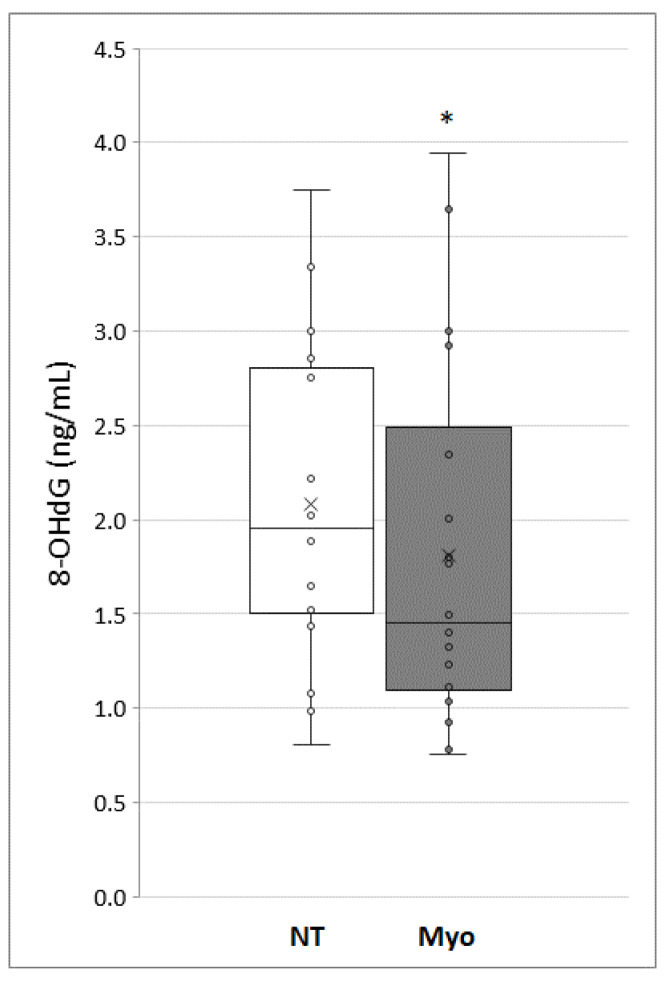
Level of oxy-DNA, measuring the 8-Hydroxy-2′-deoxyguanosine (8-OHdG) level, expressed in ng/mL, in treated and untreated (NT) sample with myo-inositol (Myo). The graph shows the normalized average oxy-DNA expressed, the standard deviation and the statistical significance of the data (*p < 0.05).

In order to evaluate the ability of myo-inositol to counteract the oxidation damage to DNA, we measured 8-Hydroxy-2′-deoxyguanosine (8-OHdG) level in sperm untreated or treated with myo-inositol 20 mg/mL for 30 min. As shown in Figure 4, a significant decrease in 8-OHdG was demonstrated in sperm treated with myo-inositol. Being 8-OHdG a specific quantitative marker of oxidative DNA damage, our data demonstrate that myo-inositol in vitro treatment could have a key role in protecting sperm from oxidative stress.

## 4. Discussion

To the best of our knowledge, this study represents the first complete evaluation of the mechanisms induced in human sperm by myo-inositol in vitro treatment. First of all, here we confirm that in vitro supplementation of myo-inositol is able to significantly improve sperm motility in a dose-dependent manner, when compared to placebo supplemented media. In particular, treatment with myo-inositol of uncapacitated sperm increases progressive motility of up to 13% (*p* < 0.05), and interestingly, the addition of myo-inositol before swim-up significantly increases the number of recovered sperm as well as their progressive motility, suggesting this treatment as recommended to improve in vitro capacitation rescue.

Several studies have reported the efficacy of oral myo-inositol administration in patients with idiopathic infertility in ameliorating main sperm parameters such as number, concentration and motility [29,32,40].

Moreover, other studies proved that in vitro incubation with myo-inositol increases significantly progressive and total motility in normozoospermic and in oligo-astheno-teratozoospermic men [30,32,33,41,42]. This protective activity exerted by inositols appears to be due to its antioxidant properties: in fact, myo-inositol has been listed among the antioxidants able to ameliorate main sperm parameters, even if the mechanisms underlying this activity are still now largely unknown.

Due to the lack of cytoplasmic defenses and to the high concentration of polyunsaturated fatty acids in their plasma membrane, sperm are very susceptible to oxidative stress triggered by increased concentration of ROS levels. Noteworthily, the damage to the mitochondrial membrane induced by high levels of ROS may compromise ATP synthesis that, consequently, impairs sperm function, such as viability and motility [36,38,43]. Moreover, a vicious circle is established, since the damage to the plasma membrane induces, in turn, an increase in the concentration of ROS [44]. To this regard, the main strength of the present study relies on the use of oxygraphic analysis as a powerful strategy to measure mitochondrial activity. In this way, not only do we confirm the positive effect of myo-inositol on mitochondria, but, for the first time, we provide clear evidence that this powerful antioxidant specifically acts by increasing OXPHOS efficiency, both in basal condition and, to a greater extent, in in vitro capacitation. Indeed, to date, only indirect evidence has been reported, as the increase in mitochondrial membrane permeability highlighted by immunofluorescent staining with the marker JC-1 [34,45].

Our data, evidencing a more efficient OXPHOS in treated sperm, are in agreement with the current knowledge on molecular pathway elicited by myo-inositol. Indeed, it has been reported that this compound, by increasing cytosolic and mitochondrial calcium levels, may make oxidative mechanisms more effective, thus ameliorating sperm mitochondrial function, preventing apoptosis, and facilitating chromatin compactness [30]. Here, we provide evidence for a significant increase of oxygen consumption in in vitro selected sperm compared to sperm in basal condition. This may be explained by the observation that more than a higher percentage of swim-up selected sperm shows a rapid progressive motility, when compared to basal samples. Sperm motility is sustained by a continuous production of ATP from the mitochondrial respiratory chain, strictly coupled to ATP synthase, that is much more efficient in energy production than glycolysis [38]. Therefore, our study definitively confirms that the demonstrated capability of myo-inositol to significantly improve sperm motility is related to its in-vitro ability to improve sperm mitochondrial function.

Moreover, our in vitro study provides significant information that, if properly translated into the clinical practice, may improve procedures currently used in ART laboratories, in order to ameliorate in vitro fertilization (IVF) protocols. Indeed, it is well known that oxidative stress increases during in vitro sperm selection procedures, consequently increasing DNA damage [46,47], thus OS represents a real threat not only for the fertilization rate, but also for the health of the offspring. Indeed, bypassing all natural sperm selection strategies, assisted fertilization techniques significantly increase the risk that sperm with damaged DNA can fertilize the oocyte [48].

To this regard, another strength point of this study was the definitive demonstration that myo-inositol supplementation in media used for semen selection can significantly protect DNA from oxidative damage during the in vitro sperm selection procedures. The 8-OHdG, one of the early products of oxidative DNA damage [23] was measured in present study, clearly demonstrating a significant decrease in this oxidative stress marker in the samples treated with myo-inositol. The inverse relationship, demonstrated in this study, between oxidative DNA damage and myo-inositol treatment is consistent with previous studies reporting the existence of a close correlation between 8-OHdG levels and sperm morphology and functionality [26,49,50], further supporting the potential value of myo-inositol use in the routine of IVF laboratory.

Finally, interesting insights come from the ultrastructural evaluation of sperm treated with myo-inositol. Here, we provide evidence that this antioxidant is able to reduce the presence of amorphous fibrous material around sperm and we confirm a previous study demonstrating that myo-inositol is able to reduce mitochondrial cristae damage [34]. Therefore, we may hypothesize that the significant increase in sperm motility that we demonstrated both in basal and in capacitated sperm may be due to ability of myo-inositol to reduce the viscosity, by removing this amorphous material.

The systematic methodological approach set up in this study allows to overcome the incomplete results still now presented in the literature, thus clarifying the pivotal role of antioxidant strategies in ameliorating sperm motility and OXPHOS efficacy. Although further studies are needed to fully understand the mechanism of action of myo-inositol, these results confirm its effectiveness in ameliorating sperm parameters during in vitro selection procedures and demonstrate that this compound specifically acts at the mitochondrial level, where it exerts positive effects on oxidative phosphorylation process and ATP production, thus explaining the improvement of sperm motility. With the increasing awareness and understanding of the protective effect of myo-inositol on sperm provided in this study, the development of evidence-based guidelines, aiming to make more effective the in vitro procedure currently used in ART laboratory for sperm selection, seems to be crucial. Indeed, protecting sperm against damage from ROS will be an important tool to minimize the risk that sperm with damaged DNA can be included in the fertilizing pool of sperm.
